# Recognition System Using Fusion Normalization Based on Morphological Features of Post-Exercise ECG for Intelligent Biometrics

**DOI:** 10.3390/s20247130

**Published:** 2020-12-12

**Authors:** Gyu Ho Choi, Hoon Ko, Witold Pedrycz, Amit Kumar Singh, Sung Bum Pan

**Affiliations:** 1 IT Research Institute, Chosun University, Gwangju 61452, Korea; ghchoi@chosun.kr (G.H.C.); skoh21@chosun.ac.kr (H.K.); 2 Department of Electrical and Computer Engineering, Alberta University, Edmonton, AB T6G 2R3, Canada; wpedrycz@ualberta.ca; 3 Department of Computer Science Engineering, National Institute of Technology Patna, Patna 800005, India; amit.singh@nitp.ac.in

**Keywords:** biometrics, user identification, post-exercise ECG, normalization, P wave, T wave, linear interpolation

## Abstract

Although biometrics systems using an electrocardiogram (ECG) have been actively researched, there is a characteristic that the morphological features of the ECG signal are measured differently depending on the measurement environment. In general, post-exercise ECG is not matched with the morphological features of the pre-exercise ECG because of the temporary tachycardia. This can degrade the user recognition performance. Although normalization studies have been conducted to match the post- and pre-exercise ECG, limitations related to the distortion of the P wave, QRS complexes, and T wave, which are morphological features, often arise. In this paper, we propose a method for matching pre- and post-exercise ECG cycles based on time and frequency fusion normalization in consideration of morphological features and classifying users with high performance by an optimized system. One cycle of post-exercise ECG is expanded by linear interpolation and filtered with an optimized frequency through the fusion normalization method. The fusion normalization method aims to match one post-exercise ECG cycle to one pre-exercise ECG cycle. The experimental results show that the average similarity between the pre- and post-exercise states improves by 25.6% after normalization, for 30 ECG cycles. Additionally, the normalization algorithm improves the maximum user recognition performance from 96.4 to 98%.

## 1. Introduction

Recently, security technology is evolving along with artificial intelligence technology. From physical security to security using software, and by recognizing bio-information, it is moving toward simple and personalized security without risk of loss [1,2,3]. External environment security technology using bio-information gradually requires personal identification in a non-face to face method, and internal environment security technology carried by users is being carried out in portable smart devices and body-wearing wearable devices [4,5,6]. Biometrics technology that authenticates individuals using bio-information was initially mainly used in the access control area, but as it was applied to service and solution authentication, it was applied to electronic finance, information communication, medical care, social welfare, administration, immigration, and entertainment. It is being used in various fields [7,8,9]. 

Biometrics is a technology for registering and recognizing individual physical and behavioral characteristics through real-time analysis [10]. Physical characteristics include external information of the body such as fingerprint, face, iris, voice, and veins of the back of the hand, and behavioral characteristics include external signals of the body such as voice, gait, handwriting, keyboarding, signature, and internal signals of the body such as the electrocardiogram (ECG), electromyography (EMG), and the electroencephalogram (EEG), etc. Biometrics using external signals has a problem in that a high error rate occurs due to lower recognition performance than in bio-information when a user authenticates it [11]. Biometrics technology using bio-information based on physical characteristics is analyzed with high recognition performance but has been a social issue due to forgery and alteration events and accidents [12,13]. For example, in a financial incident in South Korea, fake silicon fingerprints were fabricated using a 3D printer. Fake fingerprints were used in an e-passport incident at an international airport in Japan. In a hacking incident, a German hacker group duplicated the iris of the Russian President using his picture. All these incidents reveal the negative aspects of biometrics, which have become social issues. Accordingly, major developed regions such as the United States, Europe, and Japan, have been researching and developing biometric systems using bio-signals that exist within the body [14,15,16,17,18].

The ECG, a representative bio-signal inside the body, is unique to each individual, owing to the electrophysiological factors of the heart, as well as its location, size, and physical condition. However, because it is an electrical signal, it is affected by behavioral features and varies according to the measurement environment [19]. Particularly, post- and pre-exercise ECGs do not coincide, because of the temporary tachycardia, which can degrade the user recognition performance. Although studies on normalization have been conducted to match post- and pre-exercise ECGs, they experience problems such as distortions of the P wave, QRS complexes, and T wave, which are morphological features as well as unique biometric information.

The post-exercise ECG cycle is a problem that is not matched with the pre-exercise ECG cycle, and the recognition performance is degraded. In this paper, we propose a method that combines the time and frequency normalization method, and recognition system using an optimized classifier Long Short Term Memory (LSTM) based on grid search. We aimed for a fusion method to match the post-exercise ECG cycle with the pre-exercise ECG cycle in consideration of the morphological features of the ECG cycle. The time normalization method extends the P and T wave sections of the post-exercise ECG cycle by data linear interpolation and is consistent with the pre-exercise ECG cycle [1]. The frequency normalization methods find a matching optimal band in the frequency spectrum and match it to one pre-exercise ECG cycle using a bandpass filter [20]. After time normalizing the post-exercise ECG using the normalization algorithm, the average similarity is improved by 23.5% for 30 ECG cycles. By fusion normalization, the average similarity was confirmed to be 25.6% excellent performance. The proposed system was analyzed better than other classifiers by an average of 5% (SVM), 7% (K-NN), and 9% (Auto-encoder). The maximum user recognition performance of the frequency normalization algorithm, based on LSTM is 94.8%, whereas that of the time normalization algorithm is increased to 96.4%. Besides, the fusion normalization algorithm is increased to 98%. In this paper, related studies are introduced in Section 2, and the time normalization method and user recognition system are presented in Section 3. The experimental method, experiment results, and future research directions are discussed in Section 4, and the conclusions are drawn in Section 5, stressing the originality of this study.

## 2. User Recognition Technique Using Normalized ECG

**Figure 1:**
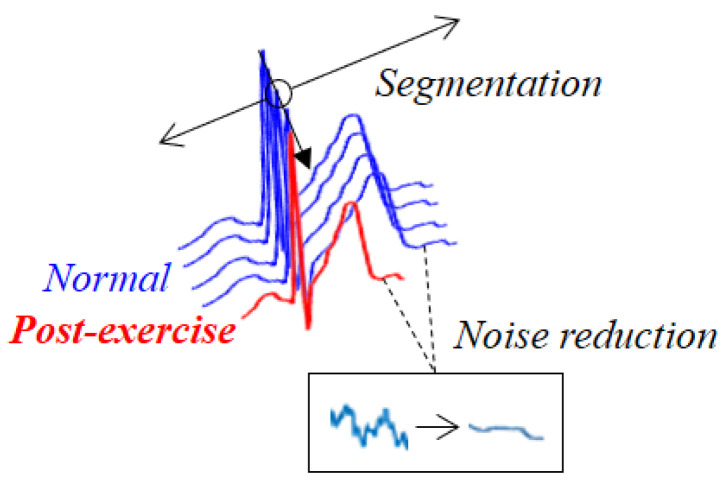
ECG in normal and post-exercise states, after preprocessing.

The technical organization of a user recognition system that uses ECG includes the creation of a database (DB) using the measured ECG, signal processing (preprocessing) to remove noise from the original ECG signal, segmentation based on the fiducial and non-fiducial points, feature extraction and reduction of feature vector dimensions in the segmented areas, and recognition of users using classification results predicted by classifiers. Figure 1 shows ECG cycles in which noise has been removed, and the P wave, QRS complexes, and T wave have been divided into morphological features in the normal and post-exercise states for normalization during preprocessing. The tachycardia ECG cycle that temporarily occurs in the post-exercise state, and not in the relaxed state, does not coincide with the ECG cycle in the pre-exercise state, which degrades the recognition performance. To improve the recognition performance, normalization is performed to match the post- and pre-exercise ECG.

### 2.1. Normalized ECG in Normal State

ECG in the normal state is used in existing user recognition systems that incorporate ECG. The recognition performances using the normalization methods proposed in various preprocessing activities are presented in Table 1.

A normalization method that excludes the ECG cycles that do not match the enrolled ideal ECG, through cross-correlation analysis is proposed in [21]. A normalization method for resampling the data of each section of the P wave, QRS complexes, and T wave of one ECG cycle is proposed in [22]. A correction process using the valley of the P wave and peak of the R wave, a process of normalizing the data of each section of the P wave, QRS complexes, and T wave of the ECG signal, and a normalizing method with one cycle that is passed by similarity with the enrollment data in each section are proposed in [23]. Normalization through segmentation by setting one ECG cycle is conducted in [24]. Normalization that segments the P and T sections, based on the R wave is conducted in [25]. Normalization is performed with 360 data samples based on the R wave, in [26]. The ECG normalization method in the normal state considers only noise removal and morphological features. It does not consider normalization for tachycardia ECG that may generally occur in the post-exercise state.

### 2.2. Normalized ECG in Post-Exercise State

ECG signals are characterized by behavioral features and are measured differently depending on the subject’s condition. Therefore, they are measured in the relaxed state as well as in other states and are analyzed by the user recognition systems after normalization. A method of measuring ECG while the subject was lying, sitting, crouching, or standing, followed by normalizing the ECG cycle for each state using a discrete wavelet transform (DWT) was proposed in [19]. Particularly, studies that apply normalization methods to each region of the ECG measured during the tachycardia, which temporarily occurs after exercise, and analyze the recognition performance have been conducted, as shown in Table 2. The ECG measured in the post-exercise state after performing jumping jacks and push-ups was normalized based on the R wave [27]. For the ECG measured in the post-exercise state after the stepper exercise, the fiducial point-based QRS complexes were normalized [28]. An optimized bandpass filter (OBPF) method was proposed for normalizing the ECG measured when the subject was in the post-exercise state after raising a foot, using a bandpass filter after selecting the optimal frequency band [20]. A method of normalizing the ECG measured, in the phase domain, when the subject was in the post-exercise state after the upright magnetic bike exercise was proposed in [29].

In terms of the user recognition performance when using post-exercise ECG, all methods except for the OBPF normalization method exhibited performance lower than that of the methods using the ECG obtained in the relaxed state. The OBPF normalization method that exhibited high recognition performance was analyzed using a few subjects. Both the OBPF normalization and phase domain normalization methods distort the morphological features of one ECG cycle. The time-domain normalization methods use only the QRS complexes, which are not affected in the post-exercise tachycardia ECG, instead of the morphological features of the P and T waves, and thus, can distort information unique to individuals.

This study proposes a time-domain normalization method that matches the post-exercise ECG to the pre-exercise P wave, QRS complexes, and T wave, and maintains the morphological features, similar to the ECG obtained in the relaxed state that exhibits high recognition performance. The normalization method uses the OBPF frequency normalization method, which has one of the highest recognition performances among the existing normalization methods. The recognition performances were compared and analyzed.

## 3. User Recognition System Using Normalized ECG Based on P and T Wave Linear Data Interpolation

**Figure 2:**
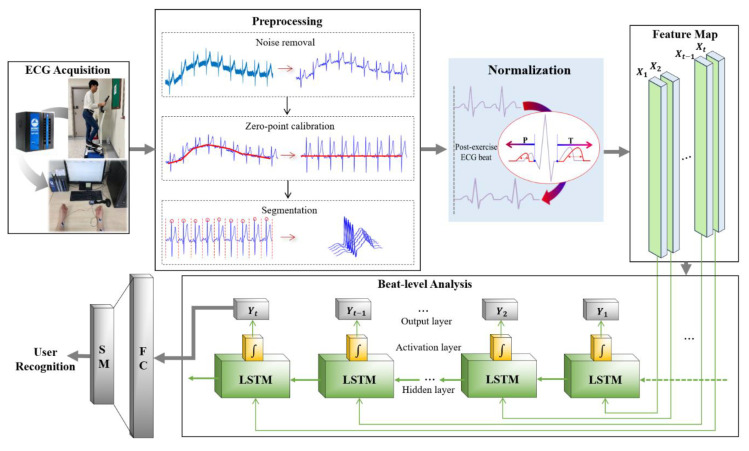
User recognition system using normalized ECG.

Figure 2 shows a flow chart of the user recognition system that uses pre-exercise and post-exercise ECG as enrollment and recognition data, respectively. The system includes the ECG lead-I acquisition process, signal preprocessing process, one cycle segmentation process, normalization process using the linear data interpolation of the P and T-wave sections, and the user recognition process.

### 3.1. User Recognition System

The ECG lead-I is measured from the subject using a measuring instrument, based on the standard 12-lead electrocardiography. The enrollment and recognition data are created by acquiring the ECG from the subject before and after exercise. Noise in the measured ECG is removed through the frequency filtering process, R wave peak detection process, and a median process excluding the QRS complexes. Noise in the ECG is removed by applying the Butterworth bandpass frequency filter. The R wave peak of the ECG that passes through the bandpass filter is detected using the threshold value of the Pan&Tompkins (1985) algorithm [30]. The detected R wave peak is used to set the QRS complexes for the application of the median filter. The QRS complexes are excluded from the median filter section because they contain biometric information unique to each individual.

Although the noise is removed using frequency filtering and the median filter, the baseline drift caused by the subject’s breathing is not removed. To remove this baseline drift, zero-point adjustment is performed using continuous first order regression analysis, for minimizing morphological feature distortion [31]. For the ECG, a periodic signal, the domain used for user recognition must be segmented. ECG’s segmentation is classified into the fiducial point and non-fiducial point segmentation. Fiducial point segmentation, which uses the morphological features of the ECG, exhibits higher recognition performance than non-fiducial point segmentation [32]. In this study, to segment one cycle based on non-fiducial point segmentation, a cycle is set from 0.2 s to the left of the R wave peak detected by the Pan&Tomkins algorithm, which corresponds to the P wave section, to 0.4 s to its right, which corresponds to the T wave section. The normalization process performs resegmentation of the segmented cycle, based on the P and T waves, and extends the P and T wave sections reduced by tachycardia by performing linear interpolation for empty data [1]. For feature extraction using the normalized ECG, all the data of one ECG cycle are selected as the morphological features. The classification for the final user recognition is performed using classifiers.

### 3.2. Linear Interpolation Based Normalization of P and T Wave Section Data in Post-Exercise ECG

Post-exercise ECG has more heartbeats than pre-exercise ECG for the same period because of the temporary tachycardia. For post-exercise, the P and T wave sections are contracted because the periodic P wave, QRS complexes, and T wave occur relatively sooner than in the pre-exercise ECG. The QRS complexes are identical before and after exercise and are unaffected by tachycardia.

**Figure 3:**
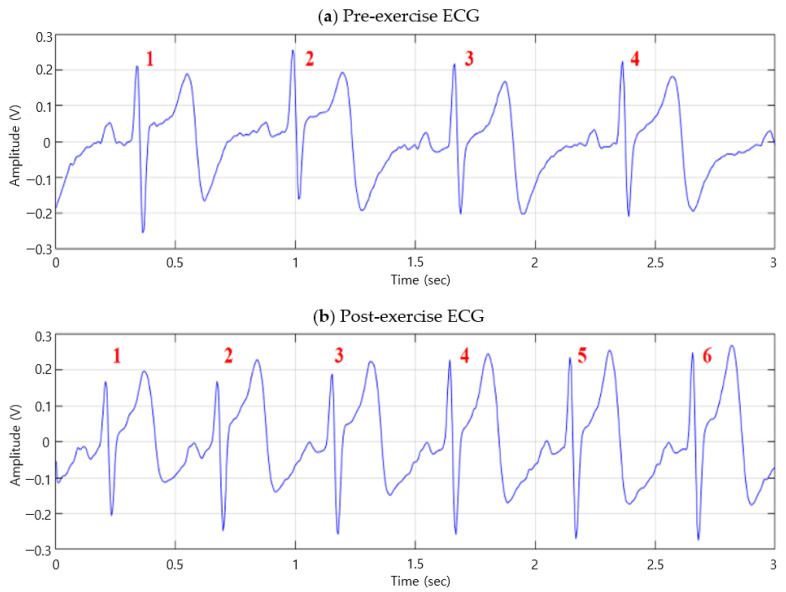
(**a**) Pre-exercise ECG (**b**) Post-exercise ECG.

**Figure 4:**
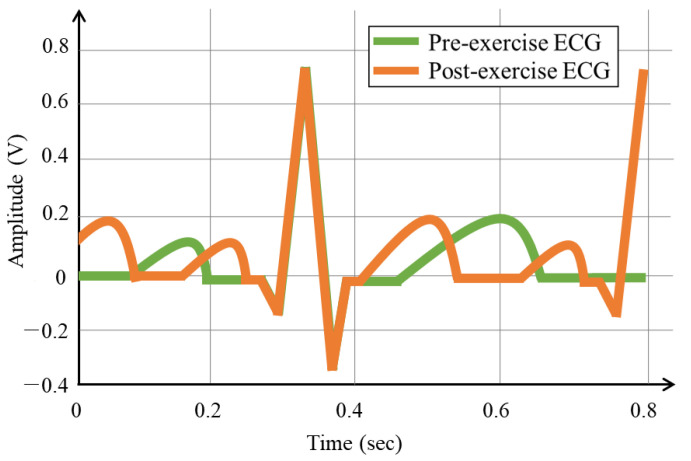
ECG segmented signals based on the R wave peak in pre- and post-exercise cases.

Figure 3 shows the ECG signal graphs before and after exercise. The ECG cycle before exercise occurs four times in three seconds, as shown in Figure 3a, whereas the ECG cycle after exercise occurs six times in three seconds, as shown in Figure 3b. This verifies that post-exercise ECG has more cycles than pre-exercise ECG, because of the temporary tachycardia. Figure 4 shows the ECG cycles, before and after exercise, segmented based on the R wave peak. As the number of ECG cycles in a period increases after exercise, segmentation was based on the R wave peak results in contracted P and T waves, compared to that of the pre-exercise ECG, and the detection of the next R wave. The segmented tachycardia ECG cycles after exercise do not coincide with the ECG cycles before exercise, because of the detection of the contracted P and T waves and next R wave.

**Figure 5:**
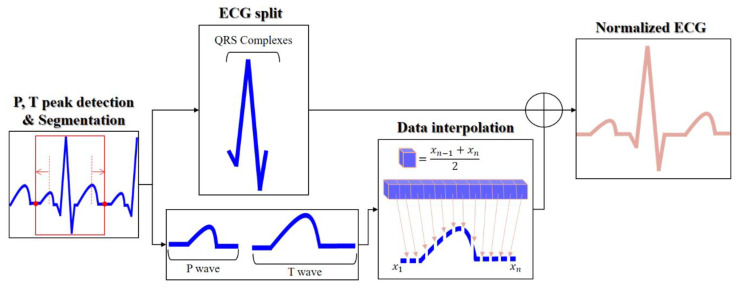
Block diagram for normalization based on P and T waves.

To match the post-exercise and pre-exercise ECG cycles, the normalization method performs linear data interpolation for the split P and T waves, as shown in Figure 5. The normalization process consists of the P and T wave peak detection, P and T wave-based cycle resegmentation, wave splitting, linear data interpolation for the P and T wave sections contracted by tachycardia, and normalization by combining the split waves.

The P wave generated by the depolarization of the atria is the section to the left of the R wave peak, and the maximum value of the P wave is detected as the maximum value in the P wave region. The T wave generated by the depolarization of the ventricles is the section to the right of the R-wave peak, and the maximum value of the T wave is detected as the maximum value in the T wave region. One cycle resegmentation is set from $P_{l}$s, which is on the left side of the P wave peak, to $T_{r}$s, which is on the right side of the T wave peak. From the P wave, QRS complexes, and T wave, each wave section is split into subsections. The P wave section is set from $P_{l}$s, which is on the left side of the P wave peak, to $R_{l}$s, which is on the left side of the R wave peak. The QRS complexes are set from $R_{l}$s, which is on the left side of the R wave peak, to $R_{r}$s, which is on its right side. The T wave section is set from $R_{r}$s, which is on the right side of the R wave peak, to $T_{r}$s, which is on the right side of the T wave peak.

**Figure 6:**
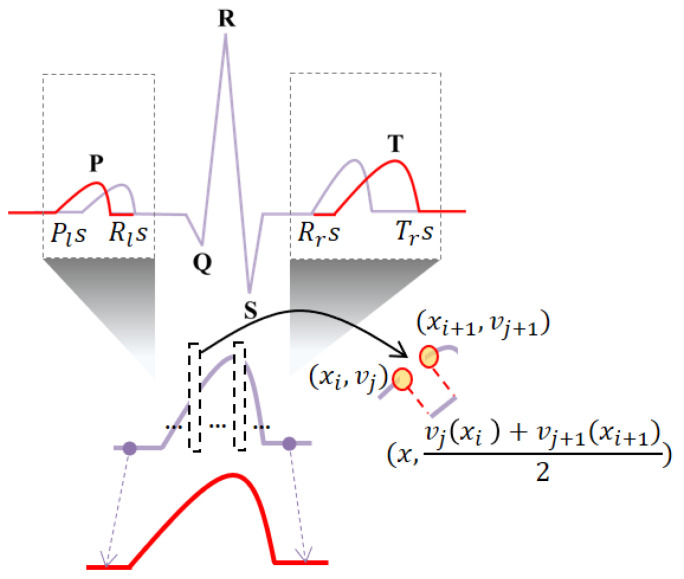
Normalization of P and T waves through linear interpolation.

The linear data interpolation of the P and T waves is performed using interpolation count, position, and amplitude calculations, as shown in Figure 6. The interpolation count $N_{i}$ is calculated as the difference between the normalized data of the P and T waves $d_{n}$ and actual data $d_{r}$ using (1).

(1) $$d_{n}=\;r_{s}\times t_{n}\;d_{r}=\;r_{s}\times t_{r\;}\;N_{i}=\;d_{n}-d_{r}$$

The normalized section data $d_{n}$ are calculated as the products of the duration of each of the P and T waves normalized based on the morphological features, $t_{n}$, and the data sampling rate, $r_{s}$. The actual data $d_{r}$ are calculated as the products of the duration of each of the actual P and T waves $t_{r}$ and the data sampling rate $r_{s}$.

(2) $$x=\;Int\left(\frac{d_{r}}{N_{i}+1}\right)\times i+1,\;i=1,\;\ldots ,\;N_{i}\;$$

The interpolation position, *x*, is calculated as an integer, using the interpolation count $N_{i}$ and the actual number of data $d_{r}$, as shown in Equation (2). *x* is generated after shifting by +1 because it is generated at a new position $x_{i}$.

(3) $$v=\frac{v_{j}\left(x_{i}\right)+v_{j+1}\left(x_{i+1}\right)}{2}\;$$

The interpolation amplitude, *v*, is calculated as the average of the magnitudes of the voltage data on the left and right sides of the interpolation position *x*, using (3). In the frequency normalization method, the ECG pre- and post-exercise in the frequency spectrum is equally performed with an optimal bandpass filter. The optimal frequency band is analyzed equally from 10 Hz to 80 Hz as a result of analyzing ECG signals pre- and post-exercise in the frequency spectrum. Therefore, the ECG signal pre- and post-exercise is normalized by a bandpass filter from 10 Hz to 80 Hz. In the fusion method, the pre-exercise ECG signal is only normalized as the frequency method, and the post-exercise ECG is normalized as time method and then normalized as frequency to match the pre-exercise ECG.

**Figure 7:**
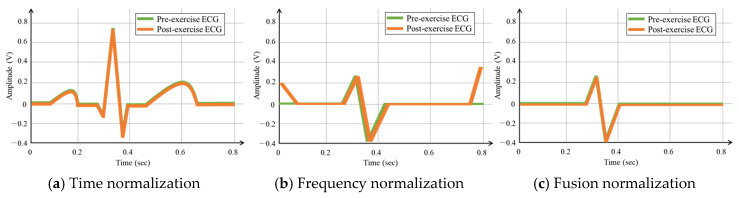
ECG signal cycle in pre and post-exercise cases as time, frequency, and fusion normalization.

Figure 7 shows the post-exercise ECG cycle that coincides with the pre-exercise ECG cycle, generated through the combination of the time, frequency normalization. Unlike the case in Figure 4 before normalization, the ECG cycles pre- and post-exercise coincide in the normalization method. The fusion normalization method produces the P wave, QRS complexes, and T wave with the same cycles as the ECG in the normal state, which exhibits high recognition performance while maintaining the morphological features.

## 4. Experiment Results

An experiment to analyze the normalization method and its user recognition performance was performed using Matlab R2019a running on a personal PC with an Intel Core i7 processor. The number of subjects that participated in the ECG measurement for the experiment was 100–64 males and 36 females, of ages ranging from 23 to 34; all of them were associated with the Chosun University. The ECG measurement protocol is presented in Table 3.

**Figure 8:**
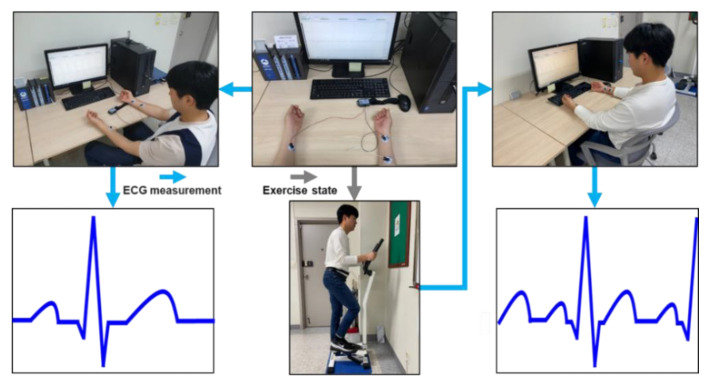
Conditions for pre- and post-exercise ECG measurement and the morphological features of the measured ECG one cycle. The cycles before and after exercise and compare the user recognition performances before and after normalization.

The ECG measurement was performed three times for each subject at 2–3 day intervals. It was measured for 60 s before exercise and 180 s after exercise in a comfortable sitting posture as shown in Figure 8. To measure the post-exercise ECG, the subject exercised with a stepper for 180 s with attached electrodes. The post-exercise ECG was measured immediately after the exercise, in a sitting posture. The ECG obtained from the measuring instrument MP160 was lead-I ECG, which could be acquired from both wrists using wet electrodes at a sampling rate of 2000 Hz based on the international standard 12-lead electrocardiography. An experiment for evaluating the normalization method using the measured ECG data was conducted to analyze the average similarity from the ECG

For the similarity evaluation, the average similarity with and without normalization in the pre-exercise state was analyzed. The average similarity with and without normalization in the pre- and post-exercise states was also analyzed. The number of cycles used in the similarity analysis was increased to 30, in five cycles for each subject. To evaluate one ECG cycle similarity before and after exercise, the Euclidean distance was calculated using Equation (4).

(4) $$d\left(p,\;q\right)=\;\sqrt{\overset{n}{\underset{i=1}{\sum }}{\left(q_{i}-p_{i}\right)}^{2}}$$

**Figure 9:**
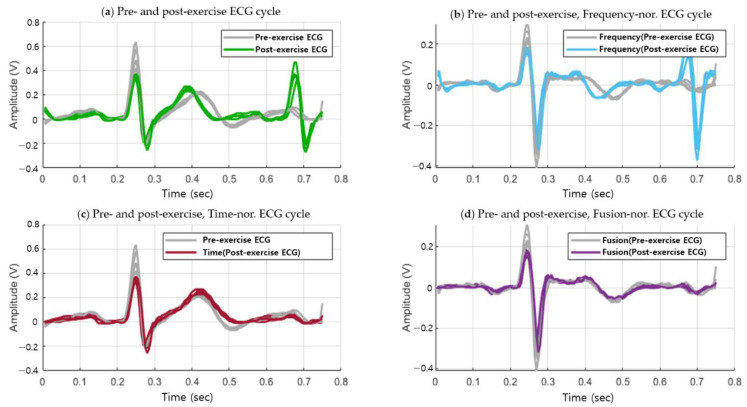
(**a**) ECG cycles before and after exercise (**b**) ECG cycles before and after exercise: existing normalization (**c**) ECG cycles before and after exercise: time normalization (**d**) ECG cycles before and after exercise: fusion normalization.

To compare and analyze the average similarity performances, the OBPF was applied using the existing normalization method, as shown in Figure 9b, by the time normalization method, as shown in Figure 9c, and by a fusion method that combined the time and existing methods, as shown in Figure 9d. The existing method analyzed the ECG cycles before and after exercise using the frequency spectrum and normalized them by finding the matching band and applying it in the same manner.

When all the data from one cycle were used for comparing and analyzing the average similarity according to the number of ECG cycles for each subject, the average similarity of the subjects was obtained as presented in Table 4. The application of the normalization method to 20 subjects improved the average similarity by 1.2% and 23.5% in the pre-exercise and post-exercise states, respectively. The application of the existing OBPF normalization method improved the average similarity by 0.92% and 13.9% in the pre-exercise and post-exercise states, respectively. The application of the fusion method improved the average similarity by 1.6% and 25.6% in the pre-exercise and post-exercise states, respectively. Regardless of the state, the average similarity increased gradually as the number of ECG cycles increased, both before and after normalization. The average similarity increase rates in the pre- and post-exercise states after normalization were significantly higher than those in the pre-exercise state. Particularly, the time normalization method exhibited a higher average similarity increase rate than the existing method, and the fusion method exhibited the highest average similarity increase rate. This is because the post-exercise ECG signals were more consistent with the pre-exercise ECG cycles because the time normalization method performed normalization in the time domain following which the existing method performed normalization in the frequency domain. The time normalization method increased the average similarity of the existing method and solved the problem of low average similarity between ECG cycles in the pre- and post-exercise states.

The user recognition performance was measured using an accuracy calculation method. The accuracy is a measure that represents the degree of prediction and matching of the recognized subject class from the total enrolled subject class. The precision, recall, F1 score, accuracy is calculated using true positive (TP), true negative (TN), false positive (FP), and false-negative (FN) values, which are predicted by 1:N matching, as follows:

(5) $$Precision=\frac{TP}{TP+FP}\;$$

(6) $$Recall=\frac{TP}{TP+FN}\;$$

(7) $$F1\;Score=2\times \frac{Precision\times Recall}{Precision+Recall}\;$$

(8) $$Accuracy=\frac{TP+TN}{TP+FN+FP+TN}\;$$

The ECG data acquired in the pre-exercise state were used as enrollment data and those acquired in the post-exercise state were used as recognition data. The classification performance of the recognition data from the enrollment data was subjected to dimensionality reduction by discriminant analysis before and after normalization and was analyzed according to the ECG cycle using various classifiers. 

In this paper, to compare and analyze the performances of various classifiers used in the time normalization method, the recognition performance was analyzed using the K-NN classifier, support vector machine (SVM, among machine learning), and auto-encoder and LSTM, which are deep learning technologies. K-NN is a method of classifying recognition data using the k the closest enrollment data in a feature space. SVM is a method of modeling learning data by finding nonlinear boundaries in a mapped space by various kernels and classifying recognition data. The auto-encoder is a neural network that performs a dimensional reduction of the input data using a non-supervised learning method and restores them as the output. LSTM is a learning technology that protects and controls long time series data with gates and cells added in a recurrent neural network structure that repeats and maintains previous data. Table 5 shows information that parameters were set by the grid search based heuristic method. 

**Figure 10:**
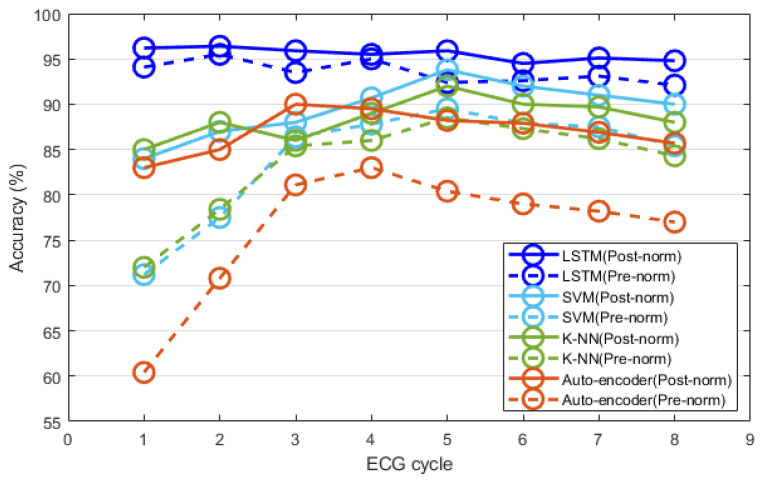
Comparative analysis of user recognition performance before and after time normalization, in terms of the number of ECG cycles.

Especially, the structure of deep learning consists of three layers of LSTM, two fully connected layers (active function: ReLU), and one output layer (active function: softmax). In the case of the fully connected layer, a dropout is applied that ignores 50% of nodes in order to prevent overfitting in which the classification algorithm is adapted to registration data. For the optimization of each network, the learning rate rmsprop was 0.001, and the registration data was evaluated by the network adjusted with 100 epochs. Figure 10 shows the user recognition performances obtained using the post-exercise ECG data measured thrice. After time normalization, the recognition performance of each classifier was compared and analyzed according to the ECG cycle. All classifiers exhibited improved recognition performances after normalization, compared to the performance before normalization. The recognition performances of the classifiers before and after normalization were generally higher when the ECG cycle number was five, compared to other ECG cycle numbers.

The classification performance of the auto-encoder reached its maximum (90%) when the ECG cycle number was three and decreased afterward. The performances of K-NN and SVM were similar, and their maximum values were 92% and 93.8%, respectively, when the ECG cycle number was five. The classification performances of K-NN and SVM were generally higher than that of the auto-encoder; however, they slowly decreased in the same manner when the ECG cycle number exceeded five. Unlike the classification performance of the other classifiers, that of LSTM reached its maximum (98.7%) when the ECG cycle number was as low as two. The recognition performance of LSTM remained similar or decreased slightly as the ECG cycle number increased. After normalization, LSTM exhibited the highest classification performance compared to all the classifiers.

To evaluate the change in recognition performance according to the increase in the number of subjects, the ECG recognition performance was analyzed after normalization. The number of subjects ranged from 20 to 100, where the number increased at intervals of 20. 

**Figure 11:**
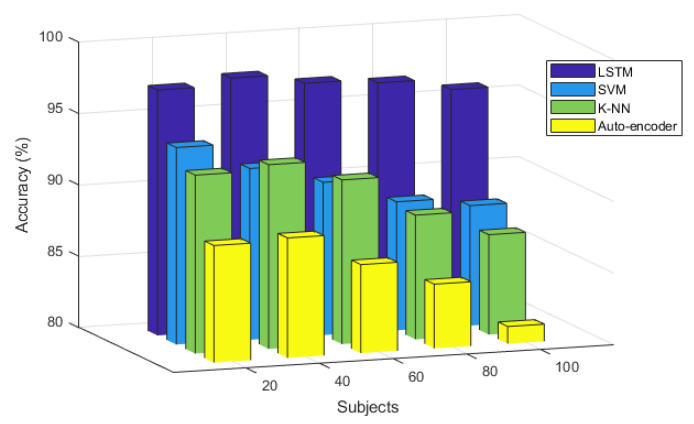
Comparative analysis of user recognition performance after time normalization, with an increasing number of subjects.

The recognition performance according to the number of subjects and classifier at ECG cycle number four, which was excellent, is shown in Figure 11. Overall, the recognition performance of the auto-encoder is the poorest and decreases slightly as the number of subjects increases; the maximum recognition performance is 88.4% when the number of subjects is 40. The recognition performances of the K-NN and SVM classifiers are similar to the number of subjects increase and are generally higher than that of the auto-encoder but lower than that of the LSTM. The recognition performance of K-NN and SVM decreased with the increase in the number of subjects as was the case in the auto-encoder. The maximum recognition performance of K-NN was 92.9% when the number of subjects was 40, whereas that of SVM was 93.8% when the number of subjects was 20. LSTM exhibited a better overall recognition performance compared to other classifiers when the number of subjects increased. It was least affected by the number of subjects and maintained the recognition performance. The maximum recognition performance of LSTM was 97.7% when the number of subjects was 60. The analysis results showed that LSTM exhibited the most outstanding recognition performance after normalization with respect to the ECG cycles before and after exercise, and with respect to the increasing number of subjects, compared to other classifiers. The recognition performance using LSTM was least affected by the cycle and number of subjects and was maintained stably.

**Figure 12:**
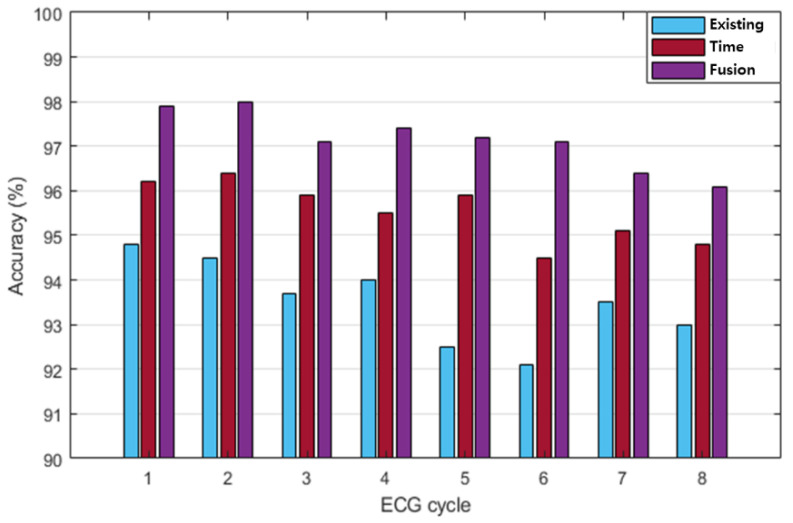
Comparative analysis of post-exercise ECG normalization recognition performance according to the number of ECG cycles (existing, time, and fusion normalization methods).

In this study, the recognition performances of existing, time, and fusion normalization methods were compared and analyzed using LSTM, which exhibited the highest recognition performance. The recognition performances were analyzed in terms of the number of ECG cycles when the number of subjects was 100. Figure 12 compares the post-exercise ECG recognition performances of each normalization method, according to the number of ECG cycles. Each normalization method exhibited the highest recognition performance when the ECG cycle number was as low as one or two. The existing method generally exhibited lower recognition performance than the time and fusion methods. The maximum recognition performance of the existing method was 94.8% when the ECG cycle number was one. Overall, the recognition performance of the time normalization method was higher than that of the existing method and lower than that of the fusion method. The maximum recognition performance of the time normalization method was 96.4% when the ECG cycle number was two. 

**Figure 13:**
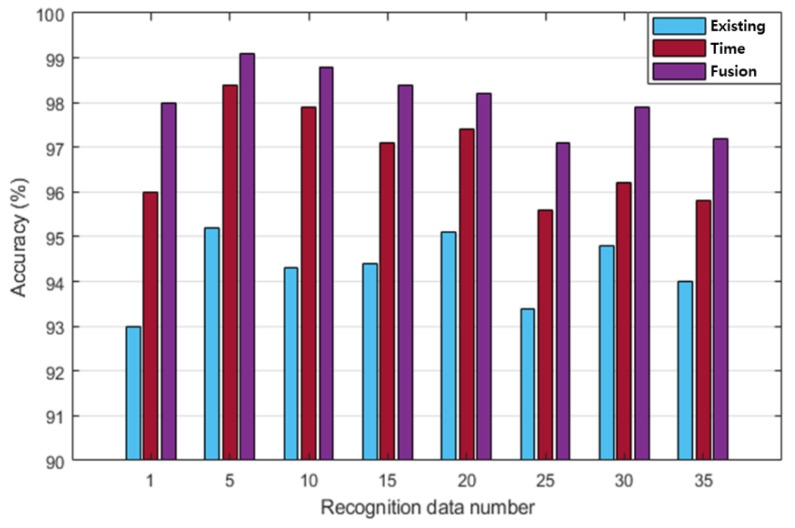
Comparative analysis of post-exercise ECG normalization recognition performance with increasing recognition data (existing, time, and fusion normalization method).

The fusion method exhibited higher recognition performance than the other methods, and its maximum recognition performance was 98% when the ECG cycle number was one or two. Table 6 shows the performance of precision, recall, F1 score, and accuracy according to each normalization method in ECG cycle two, which was analyzed as the best recognition performance. The fusion method of time and frequency normalization was best analyzed in all performance indicators. Figure 13 shows the comparison of the post-exercise ECG recognition performance according to the normalization method with respect to the change in recognition data. The recognition performance in each normalization method was not significantly affected by the changes in the amount of recognition data. The recognition performance of the existing normalization method was lower than that of the other normalization methods, with a maximum performance of 95.2% on day five of the recognition data. The recognition performance of the proposed method was higher than that of the existing method but lower than that of the fusion method. The maximum recognition performance of the proposed method was 98.4% on day five of the recognition data. Overall, the recognition performance of the fusion method was higher than those of the other normalization methods as the number of recognition data increased. The maximum recognition performance of the fusion method was 99.1% on day five of the recognition data. The analysis results showed that the recognition performance of the fusion method is the highest among all the normalization methods. The fusion method was least affected by the cycle and the increase in the amount of recognition data, whereas the recognition performance was maintained stably with a decreasing trend.

## 5. Conclusions

In this paper, in order to improve the user recognition system using post-exercise ECG, we proposed a system that analyzes it excellently like the recognition performance of pre-exercise ECG. For user recognition, this study employed the time normalization method to match the ECG after a stepper exercise, to one pre-exercise ECG cycle composed of the P wave, QRS complexes, and T wave, which were morphological features, and improved recognition performance. The user recognition system, which used normalized ECG in the post-exercise state, consisted of the ECG lead-I acquisition process, signal preprocessing for noise removal, one ECG cycle segmentation process, normalization process through the linear interpolation of the P and T wave section data in one cycle, morphological feature-based cycle feature extraction process, and classification process for user recognition.

The user recognition performance was analyzed according to the ECG cycle after exercise in 100 subjects, using the time normalization method and the database created using the ECG data measured before and after exercise. The maximum user recognition performance based on LSTM was 94.8%, 96.4%, and 98% for the frequency, time normalization, and fusion methods, respectively. Particularly, as the fusion normalization method improved the average similarity in the pre- and post-exercise states by 25.6% when the ECG cycle was 30, the post-exercise ECG cycle coincided with the pre-exercise ECG cycle. As a result of analyzing the maximum recognition performance, the frequency normalization method was 94.8%, the time normalization method was 96.4%, and the fusion normalization method was analyzed as 98%. In order to improve the user recognition system using post-exercise ECG, we proposed a system that analyzes it excellently like the recognition performance of pre-exercise ECG. When the ECG signal was acquired, normalization was performed in consideration of a single state. In order to be applied in everyday life, the normalization method is needed in complex states. In the future, the ECG will be acquired from more subjects in dynamic situations and a database will be built for the development of user authentication technology. Additionally, the database will be used for research on a user recognition system based on deep learning technology.

## Figures and Tables

**Table 1:** Preprocessing in the normal state for normalization.

ECG DB (Normal)	Noise Reduction	Segmentation	Normalization	Reference
Personal	Bandpass filter	Fiducial	Cross correlation	[21]
PTB MIT-BIH	Wavelet	Fiducial	Resampling	[22]
Personal	Bandpass filter	Fiducial	Resampling	[23]
Personal	Bandpass filter	Fiducial	Non	[24]
Personal	Cascading filter	Fiducial	Non	[25]
MIT-BIH	High-pass filter	Fiducial	Non	[26]

**Table 2:** Preprocessing in the post-exercise state for normalization.

ECG DB (Exercise)	Noise Reduction	Segmentation	Normalization	Reference
Personal (Jumping jacks and pushups)	Bandpass filter	Fiducial	Non	[27]
Personal (Stepper)	Bandpass filter	Fiducial	QRS complexes	[28]
Personal (Raising a foot)	Bandpass filter	Fiducial	OBPF	[20]
Personal (Upright magnetic bike)	Non	Non-fiducial	Phase reconstruction	[29]

**Table 3:** Electrocardiogram measurement protocol.

Times	Situation		
	Sit at Rest	Exercise (Stepper)	Sit at Rest
**1**	60 s	180 s	180 s
2–3 days break			
**2**	60 s	180 s	180 s
2–3 days break			
**3**	60 s	180 s	180 s

**Table 4:** Analysis of similarity for application of no normalization, existing normalization, time normalization, and fusion normalization for pre- and post-exercise cases.

Subject		20							
State		Pre-Exercise				Pre- and Post-Exercise			
Nor.		Non	Fre. [20]	Time [1]	Fusion	Non	Fre. [20]	Time [1]	Fusion
**E** **C** **G**	**5**	0.3777	0.3485	0.3292	0.3280	0.8827	0.6125	0.5496	0.5489
	**10**	0.2435	0.2451	0.2388	0.2374	0.7167	0.5953	0.5023	0.5015
	**15**	0.2529	0.2274	0.2252	0.2244	0.6612	0.5717	0.4731	0.4724
	**20**	0.2249	0.2197	0.2189	0.2179	0.5924	0.5384	0.4381	0.4360
	**25**	0.2263	0.2159	0.2150	0.2142	0.5437	0.4995	0.4185	0.4175
	**30**	0.2057	0.2038	0.2032	0.2024	0.5247	0.4517	0.4014	0.3903
**Average rate of similarity change**			0.92% increase	1.2% increase	1.6% increase		13.9% increase	23.5% increase	25.6% increase

**Table 5:** Hyperparameter set according to each classifier.

	SVM	K-NN	Auto-Encoder	LSTM
Kernel	rbf	-	-	-
Gamma	100	-	-	-
C	10	-	-	-
K	-	1	-	-
1-layer	-	-	100 × 8	n
2-layer	-	-	100 × 4	n/2
3-layer	-	-	100 × 2	n/3
Fully connected layer1, Drop out	-	-	-	400, 0.5
Fully connected layer2, Drop out	-	-	-	200, 0.5
Softmax	-	-	100	100

**Table 6:** Performance comparison analysis using each normalization method.

	Precision	Recall	F1-Score	Accuracy
Time nor.	0.98%	0.95%	0.97%	96.4%
Frequency nor.	0.96%	0.92%	0.94%	93.6%
Fusion nor.	0.99%	0.96%	0.98%	98%
